# Early efficacy of rTMS intervention at week 2 predicts subsequent responses at week 24 in schizophrenia in a randomized controlled trial

**DOI:** 10.1016/j.neurot.2024.e00392

**Published:** 2024-06-29

**Authors:** Suzhen Ye, Xiaoni Guan, Meihong Xiu, Fengchun Wu, Yuanyuan Huang

**Affiliations:** a Department of Rehabilitation, The First Affiliated Hospital of Wenzhou Medical University, Wenzhou, China; b Peking University HuiLongGuan Clinical Medical School, Beijing HuiLongGuan Hospital, Beijing, China; c Department of Psychiatry, The Affiliated Brain Hospital, Guangzhou Medical University, Guangzhou, China; d Guangdong Engineering Technology Research Center for Translational Medicine of Mental Disorders, Guangzhou, China; e Key Laboratory of Neurogenetics and Channelopathies of Guangdong Province and the Ministry of Education of China, Guangzhou Medical University, Guangzhou, China

**Keywords:** Schizophrenia, rTMS, Randomized controlled trial, Cognitive functions, Efficacy

## Abstract

Repetitive transcranial magnetic stimulation (rTMS) is a non-invasive brain stimulation technique for modulating cortical activities and improving neural plasticity. Several studies investigated the effects of rTMS, etc., but the results are inconsistent. This study was designed to examine whether rTMS applied on the left dorsolateral prefrontal cortex (l-DLPFC) showed an effect on improving cognitive deficits in SZ and whether the early efficacy could predict efficacy at subsequent follow-ups. Cognitive ability was assessed using the Repeatable Battery for the Assessment of Neuropsychological Status (RBANS) scale at baseline, weeks 2, 6, and 24. We found a significant interaction between time (weeks 0, 2, 6, and 24) and intervention on immediate memory and RBANS total scores (p ​= ​0.02 and p ​= ​0.04), indicating that both 10-Hz and 20-Hz rTMS stimulations had a delayed beneficial effect on immediate memory in SZ. Moreover, we found that 20-Hz rTMS stimulation, but not 10-Hz rTMS improved immediate memory at week 6 compared to the sham group (p ​= ​0.029). More importantly, improvements in immediate memory at week 2 were positively correlated with improvements at week 24 (β ​= ​0.461, t ​= ​3.322, p ​= ​0.002). Our study suggests that active rTMS was beneficial for cognitive deficits in patients with SZ. Furthermore, efficacy at week 2 could predict the subsequent efficacy at 24-week follow-up.

## Introduction

Schizophrenia (SZ) is a chronic brain disease [1]. Cognitive deficits are a core dimension of SZ, and as such there has been increasing interest in it [2,3]. Neuropsychological research has provided robust evidence for cognitive deficits in patients with SZ. Patients with SZ exhibit several domains of cognitive deficits, such as attention, verbal fluency, working memory, processing speed, declarative verbal memory, and executive functioning, which are related to poor functional outcomes frequently observed in patients [4].

Although antipsychotic medications are effective in alleviating positive symptoms of SZ, treatment efficacy is largely neutral concerning patients’ cognitive deficits [5,6]. Given the core role of cognitive deficits in patients with SZ, new interventions to address cognitive impairments are necessary. In recent years, a growing body of randomized controlled studies has investigated the use of cognitive-enhancing medications as a supplement to antipsychotic medication to improve cognitive function [7]. Non-pharmacological interventions including cognitive behavior therapy have also been reported as promising treatment options [8]. Recently, studies have focused on novel biological treatments targeting the neural mechanism of cognitive deficits and neuroplasticity in SZ [9,10].

Repetitive transcranial magnetic stimulation (rTMS) has been considered a therapy supplement to show effects on cognitive performance [11]. It is a safe, non-invasive intervention strategy hypothesized to ameliorate cognitive deficits in a variety of psychiatric disorders [11,12]. The main interest in SZ research has focused on the dorsolateral prefrontal cortex (DLPFC), a brain region in which neuropathological findings and combined evidence from structural and functional neuroimaging have revealed aberrant neuronal connectivity [[13], [14], [15]]. The DLPFC has been correlated with inhibitory control [16,17] and is involved in executive function and working memory [18]. In most of the previous literature, the left DLPFC (l-DLPFC) has been implicated as a target of rTMS [[19], [20], [21]]. rTMS plays a role in modulating cortical excitability and metabolic activity in the brain, and functional connectivity between different brain regions [[22], [23], [24], [25]]. More recently, in rTMS studies, image-guided neuronavigation systems have been used for precise targeting by co-registering patients’ images to standardized reference images [26].

rTMS has been hypothesized to improve cognitive performance in SZ. Indeed, much evidence highlights some roles of high-frequency rTMS in cognitive enhancement in healthy adults [27]. A recent review by Xu et al. reported that the rTMS treatment course produced a relatively greater effect on healthy subjects' executive functioning in accuracy and reaction time [28]. Similarly, in patients with depression disorders, rTMS demonstrated modest beneficial effects on cognition, particularly in psychomotor speed [29]. Unlike depressed and healthy subjects, the effects of rTMS on cognition have been controversial in SZ. A few pieces of literature have demonstrated the efficacy of rTMS on the domains of auditory verbal memory, working memory, and facial affect recognition domains in SZ [[30], [31], [32], [33], [34], [35]]. On the other hand, some studies did not find cognitive enhancement effects of active rTMS on verbal memory, attention, and other cognitive domains in SZ [[36], [37], [38], [39]]. Duration of rTMS intervention session, frequency of stimulations, precise targeting of DLPFC and patient characteristics could explain the discrepancy amongst studies [40,41]. Thus, the efficacy of active rTMS on cognition in SZ remains unclear and requires further investigation.

Considering the potential impact of different stimulation frequencies and intervention durations on the efficacy of rTMS, this study was designed to compare the different effects of neuronavigation rTMS on cognitive performance in patients with SZ. In addition, we explored whether the early efficacy of rTMS at week 2 could predict the responses to rTMS at week 24.

## Methods

### Subjects

This study was performed between January 5th, 2016 to December 1st, 2019. Eighty-four inpatients with a diagnosis of SZ based on the Diagnostic and Statistical Manual of Mental Disorders, Fifth Edition (DSM-5) were enrolled at Hebei Province Hospital. Written, informed consent was obtained from each patient.

Inclusion criteria included: 1) male veterans; 2) able to provide informed consent; and 3) stable dosage and type of antipsychotics in the past 24 weeks. Exclusion criteria included: 1) diagnosis of another mental illness; 2) major brain disease or physical illnesses; and 3) modified electroconvulsive therapy (MECT) treatment in the past half year.

### Procedures

**Fig. 1:**
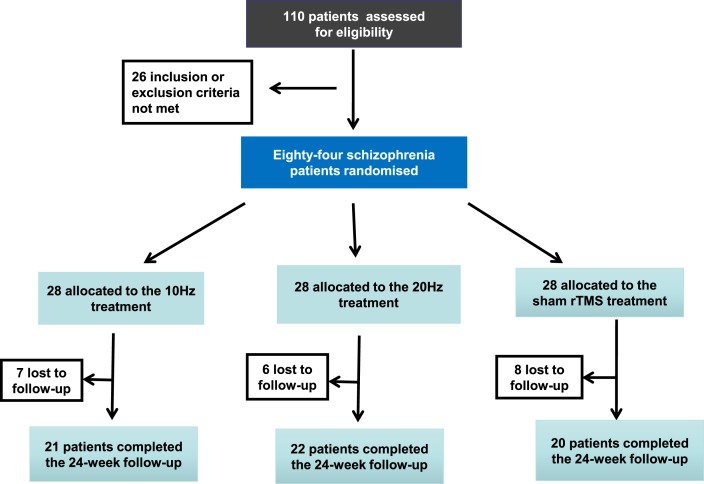
Flow chart.

This was a randomized, sham-controlled, and double-blind clinical trial. rTMS was delivered in one session per day, five sessions per week (Monday to Friday) for six consecutive weeks. The patients received rTMS for a total of 30 times. Patients were randomly allocated to the sham group (n ​= ​28), 10-Hz rTMS group (n ​= ​28), and 20-Hz rTMS (n ​= ​28) group after providing written informed consent. Investigators, clinical raters, and participators were blinded to intervention allocation. Six patients in the sham group, seven in the 20-Hz group, and eight in the 10-Hz group were lost to follow-up. The consort checklist is shown in Fig. 1.

The randomization list was produced by a computer and kept by a staff. After recruitment, the rTMS administrator allocated participants to the 10-Hz, 20-Hz, or sham group based on the randomization lists provided by the staff. The investigator and patients were blinded to treatment assignment. The staff member was the only person with access to the randomization list and had no contacts or role in measuring cognitive functions. Blinding was maintained throughout the follow-up period.

Neuronavigation-guided rTMS was applied to the left DLPFC location using the MagStim Rapid2 Stimulator. The Brainsight navigation system was used for precise targeting on l-DLPFC. The exact location of l-DLPFC has been described in a previous study [33]. The subject's head position was assessed to measure the location of the scalp markers (tip of the nose, nasion, and right and left ear invaginations) seen on MRI. Based on recent meta-analyses of functional neuroimaging studies on cognitive function, superior region Brodmann area (BA) 46 and posterior region BA9 were used as targets that the rTMS worked on. Neuronavigation software was used to estimate the root mean square of the difference between co-registered anatomical markers. After anatomical co-registration, 8-shaped coils were placed in a direction tangential to the scalp. Neuronavigation was performed on individuals' MRIs.

In the 20-Hz group, 20-Hz rTMS stimulation frequency over l-DLPFC occurred at a power of 110% of motor threshold (MT) for 2-s intervals with 28-s intervals (40 trains with 40 stimuli per train). In the 10-Hz group, 10 ​Hz stimulation frequency over l-DLPFC occurred at an intensity of 110% of MT for 3-s intervals with a 27-s interval (40 trains with 30 stimuli per train). In the sham group, the protocol was the same as in the 10-Hz group except for the sham coils. The specific sham coils looked identical to the active coils, replicating the impulse noise that stimulated the sensation of the active stimulation. Each session lasted for approximately 20 ​min. The pulses for each session in the 20-Hz rTMS group was 1600 stimuli and for the 10-Hz rTMS was 1200 stimuli.

At the end of the sixth week, rTMS treatment was discontinued. All participants were followed up at the end of the 24th week to examine the improvements in cognitive functions. In this study, only one follow-up was present 24 weeks after rTMS treatment.

No severe adverse effect associated with rTMS was found in the present study. Four patients (2 in the 10 ​Hz groups, 1 each in the 20 ​Hz and sham groups) felt dizziness during the intervention. Three patients in the active rTMS group complained of scalp pain. Seven patients (3 in the 10-Hz group, and 2 in each of the 20-Hz and sham groups) reported mild discomfort.

### Measurements

The primary outcome of this study was cognitive performance, as measured by the Repeatable Battery for the Assessment of Neuropsychological Status (RBANS) [42]. The RBANS comprises 12 subtests that are used to calculate 5 age-adjusted index scores and a total score. The test indices are immediate memory, attention, language, visuospatial/constructional, and delayed memory. It has been previously translated into Chinese and its clinical validity and test-retest reliability were established in SZ [43]. The total and index scores reported in the study were standard scores.

The secondary outcome of the present study was the severity of clinical symptoms, as assessed using the Positive and Negative Syndrome Scale (PANSS) [44]. To ensure consistency and reliability of ratings across the studies, raters simultaneously attended a training session in using the PANSS before the recruitment of participants in this study. After training, raters maintained an inter-rater correlation coefficient greater than 0.8 for the total score.

The raters assessed the clinical symptoms and cognitive functions at baseline, weeks 2 and 6, and at the 24-week follow-up. Cognitive performance and clinical symptoms were measured by the raters who had no access to the randomization lists.

### Data analysis

Individuals who dropped out were compared with those who finished the study using *X*^2^ tests and ANOVA. The missing data was imputed using the last observation carried forward (LOCF) method, with the last available visit as the endpoint. The main analyses were used on the Intent-to-Treat (ITT) basis for all patients recruited and randomized at baseline, including those who finished treatment and those who dropped out during treatment.

Demographic and clinical variables and cognitive functions at baseline were compared by *X*^2^ tests or ANOVA between the three groups. To test whether rTMS intervention for 6 weeks and follow-up for 24 weeks improved cognitive performance among three groups, repeated measures ANOVA (RM-ANOVA) was performed to compare cognitive performances over time for 10-Hz, 20-Hz, and sham rTMS interventions in eighty-four patients as the ITT analysis. For the dependent variables, 4 time points (baseline, weeks 2, 6, and 24) were entered as the RM-ANOVA within-effects, and groups were entered as the between-effects. If the interaction effect was significant, the paired sample *t*-test was performed to explore cognitive functions before and after treatment in the three groups. It is beneficial to know whether rTMS showed treatment effects in the early phase (e.g., week 2) and follow-up phase (e.g., week 24). However, it is also important to find whether the rTMS treatment showed significant effects at the end of treatment of 6 weeks. Therefore, we performed RM-ANOVA analysis to test whether rTMS intervention for 6 weeks improved cognitive performance among the three groups. For the dependent variables, 3 time points (baseline, weeks 2, and 6) were entered as the RM-ANOVA within-effects and groups (10-Hz vs 20-Hz or 10-Hz vs sham or 20-Hz vs sham) were entered as the between-effects. Differences in cognitive performances between the 10 ​Hz, 20 ​Hz, and sham groups at week 2, week 6, and week 24 were tested by ANCOVA, respectively, with the scores at baseline as covariates.

Pearson correlation analysis was performed to analyze the correlation between early efficacy at week 2 and efficacy at week 24 in the active rTMS group. Linear regression analysis was conducted to confirm the association between them, after controlling for age, educational levels, smoking status, disease duration, onset age, and times of hospitalization.

## Results

### Participant characteristics among the three groups

There was no significant difference in age, years of education, marital status, relationship with family members, smoking status, years of smoking, age of onset, duration of illness, and hospitalization time among the three groups (all p ​> ​0.05) (Table 1). We also compared the completers and dropouts and found that completers were younger, had shorter duration of illness, and had fewer times of hospitalization (p ​= ​0.009, p ​= ​0.014, and p ​= ​0.03). Also, completers had better performances in attention, delayed memory, and RBANS total scores (p ​= ​0.022, p ​= ​0.005, and p ​= ​0.003).

**Table 1:** Demographic and clinical data of active and sham rTMS groups at baseline (mean ​± ​SD).

	Sham rTMS (n ​= ​28)	20 ​Hz rTMS (n ​= ​28)	10 ​Hz rTMS (n ​= ​28)	*X*^2^ or *F(p)*
Age (yrs)	56.0 ​± ​7.3	51.9 ​± ​10.2	50.9 ​± ​8.3	2.7 (0.08)
Education (yrs)	7.8 ​± ​1.8	7.9 ​± ​2.6	7.6 ​± ​2.0	0.1 (0.87)
Marital status				0.1 (0.93)
Currently married (n)	23	22	22	
Single or divorced (n)	5	6	6	
Smoking status				0.9 (0.65)
Yes (n)	9	7	6	
No (n)	19	21	22	
Years of smoking (yrs)	20.61 ​± ​16.7	19.3 ​± ​14.7	21.5 ​± ​14.7	0.1 (0.87)
Age of onset (yrs)	21.1 ​± ​1.7	20.9 ​± ​2.4	21.3 ​± ​2.3	0.3 (0.75)
Duration of illness (yrs)	34.5 ​± ​6.9	31.3 ​± ​9.7	30.0 ​± ​8.6	2.0 (0.14)
Hospital time	5.6 ​± ​3.1	6.3 ​± ​3.2	5.8 ​± ​2.7	0.3 (0.73)
Dose of antipsychotics (mg/day, CPZ equivalents)	415.1 ​± ​211.1	413.7 ​± ​235.9	410.5 ​± ​218.5	0.02 (0.98)
Baseline symptoms				
Total score	79.9 ​± ​16.9	72.3 ​± ​12.9	70.4 ​± ​13.1	3.4 (0.04)
P subscale	11.4 ​± ​3.6	11.3 ​± ​4.5	12.2 ​± ​5.0	0.4 (0.67)
N subscale	31.9 ​± ​8.4	28.7 ​± ​6.7	27.2 ​± ​6.4	3.1 (0.05)
G subscale	36.6 ​± ​8.7	32.3 ​± ​7.6	31.0 ​± ​7.6	3.8 (0.03)

**Note:***SD* standard deviation, *yrs* years, *PANSS* Positive and Negative Syndrome Scale, *P* positive symptom, *N* negative symptom, *G* General psychopathology; *Dose of antipsychotic* (mg/day) chlorpromazine (CPZ) equivalent.

### Changes in clinical symptoms over time

We first analyzed the interaction effect of treatment and time on the severity of clinical symptoms, as assessed by the PANSS scale, and found no significant interaction on PANSS-P, PANSS-N, PANSS-G, and total scores (all p ​> ​0.05).

### Changes in cognitive performances over time

**Fig. 2:**
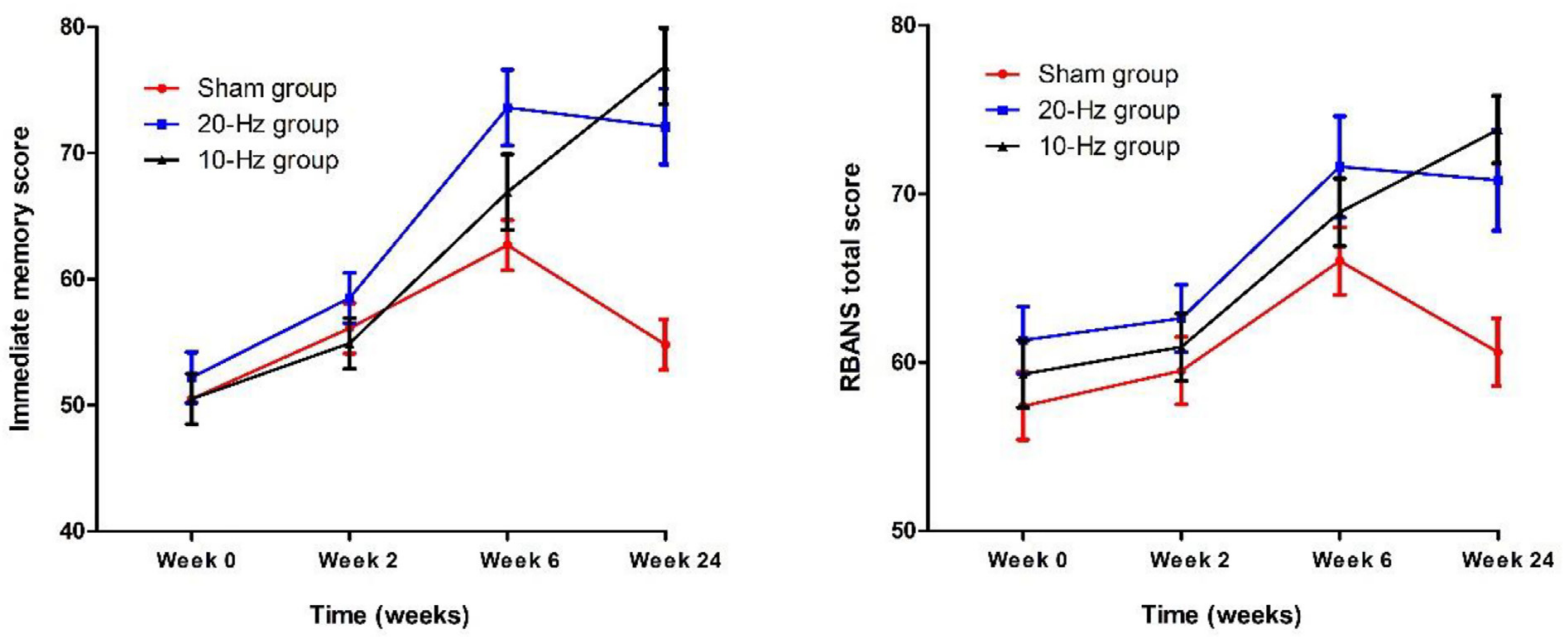
rTMS treatment significantly increased the immediate memory score and RBANS total score in the patients with schizophrenia in the active rTMS group (10-Hz and 20-Hz), relative to the sham group (p ​< ​0.05). Results are presented as a mean ​± ​standard error for all subjects.

We analyzed the cognitive performances of SZ patients among the three groups after rTMS treatment. RM-ANOVA analysis among three groups was conducted to test the efficacy of rTMS intervention on cognitive performance and showed significant interactions between time (4 time points) and intervention group on immediate memory (F_(3,84)_ ​= ​6.7, p ​= ​0.02) and RBANS total score (F_(3,84)_ ​= ​4.3, p ​= ​0.04) (Table 2) (Fig. 2). However, no significant interactions were observed between time and groups for visual-spatial, language, attention, and delayed memory index (p ​= ​0.15, p ​= ​0.10, p ​= ​0.28, p ​= ​0.11).

**Table 2:** Cognitive functions at baseline and follow-up after treatment in the three rTMS groups (mean ​± ​SD).

	Baseline	Week 2	Week 6	Week 24	Time *F(P)*	Group ​× ​Time *F(P)*
Immediate memory					58.7 (<0.001)	9.0 (<0.001)
Sham rTMS	50.5 ​± ​10.3	56.1 ​± ​17.2	62.7 ​± ​17.7	54.8 ​± ​14.1		
20-Hz rTMS	52.2 ​± ​12.5	58.5 ​± ​12.8	73.6 ​± ​23.2	72.1 ​± ​19.5		
10-Hz rTMS	50.5 ​± ​10.5	54.9 ​± ​14.3	66.9 ​± ​20.7	76.9 ​± ​19.4		
Visual-spatial					14.4 (<0.001)	1.9 (0.08)
Sham rTMS	67.9 ​± ​15.7	71.2 ​± ​17.5	72.6 ​± ​17.1	70.8 ​± ​14.7		
20-Hz rTMS	71.5 ​± ​17.0	73.4 ​± ​18.9	76.7 ​± ​18.7	80.9 ​± ​18.4		
10-Hz rTMS	70.7 ​± ​10.6	73.7 ​± ​15.0	76.3 ​± ​12.1	81.0 ​± ​15.0		
Language					6.0 (0.001)	2.3 (0.05)
Sham rTMS	73.2 ​± ​15.7	76.7 ​± ​15.7	80.9 ​± ​18.3	73.8 ​± ​14.8		
20-Hz rTMS	78.9 ​± ​35.0	79.3 ​± ​14.5	81.8 ​± ​11.9	80.9 ​± ​11.8		
10-Hz rTMS	79.3 ​± ​15.2	79.8 ​± ​13.8	84.4 ​± ​12.6	86.9 ​± ​10.4		
Attention					6.6 (<0.001)	1.2 (0.26)
Sham rTMS	65.8 ​± ​13.3	60.5 ​± ​14.2	62.6 ​± ​14.4	60.3 ​± ​12.9		
20-Hz rTMS	71.2 ​± ​15.9	65.2 ​± ​14.6	67.3 ​± ​14.7	66.3 ​± ​16.6		
10-Hz rTMS	69.6 ​± ​15.9	64.7 ​± ​17.3	66.3 ​± ​13.5	70.5 ​± ​15.2		
Delayed memory				0.03 (0.86)	44.2 (<0.001)	2.2 (0.05)
Sham rTMS	62.7 ​± ​19.9	66.4 ​± ​20.2	77.4 ​± ​22.2	70.5 ​± ​19.2		
20-Hz rTMS	64.5 ​± ​19.9	67.5 ​± ​20.4	81.4 ​± ​24.9	82.8 ​± ​21.9		
10-Hz rTMS	64.3 ​± ​16.5	67.5 ​± ​18.2	78.5 ​± ​23.6	82.0 ​± ​21.7		
RBANS total score					45.3 (<0.001)	5.0 (<0.001)
Sham rTMS	57.4 ​± ​11.8	59.5 ​± ​2.8	66.0 ​± ​15.4	60.6 ​± ​11.3		
20-Hz rTMS	61.3 ​± ​12.5	62.6 ​± ​13.6	71.6 ​± ​17.7	70.8 ​± ​16.2		
10-Hz rTMS	59.3 ​± ​9.9	60.9 ​± ​10.9	68.9 ​± ​14.1	73.8 ​± ​15.1		

**Note:***RBANS* Repeatable battery for the assessment of neuropsychological status.

To investigate the effect of 2 stimulation frequencies on cognitive function, we performed the post hoc analyses between 10-Hz rTMS vs sham, 20-Hz vs sham, and 10-Hz vs 20-Hz rTMS. We found significant differences in immediate memory (p ​< ​0.001) and RBANS total scores (p ​= ​0.03) between 20-Hz rTMS and sham, as well as between 10-Hz and sham (p ​< ​0.001; p ​< ​0.001), with no significant interaction between 10-Hz and 20-Hz rTMS (p ​= ​0.06; p ​= ​0.15) at the end of week 24. In addition, at the end of week 6, we found a significant difference between the 20-Hz and sham groups (p ​= ​0.029), with no significant interaction between the 10-Hz and sham groups or between 10-Hz and 20-Hz rTMS (p ​> ​0.05). No significant group effect was observed at baseline and the end of week 2 (all p ​> ​0.05).

Then, to investigate the effect of different time points on cognitive function, post hoc analyses were performed in sham, 10-Hz, and 20-Hz rTMS groups, respectively. We found significant improvements in immediate memory in weeks 2 and 6 in the sham group (p ​= ​0.03, p ​< ​0.001), and no difference in weeks 24 (p ​> ​0.05). In both 10-Hz and 20-Hz rTMS groups, we found significant improvements in immediate memory in weeks 2, 6, and 24 (all p ​< ​0.01). For RBANS total score, we found significant improvements in weeks 2, 6, and 24 in both 10-Hz and 20-Hz rTMS groups (all p ​< ​0.01). In the sham group, we only found significant improvements in week 6 (p ​> ​0.05).

Lastly, considering both 10-Hz and 20-Hz rTMS showed effects on cognitive functions, we combined the 10-Hz and 20-Hz rTMS groups into an active rTMS group (n ​= ​56) and further analyzed cognitive function before and after treatment. We found that relative to baseline, a significant improvement was observed in the immediate memory after treatment with active rTMS for 2 weeks (t ​= ​−3.31, p ​= ​0.002), but not in the sham group (p ​> ​0.05). ANCOVA analysis revealed that after controlling for immediate memory at baseline, the improvements between the active and sham rTMS groups were significant after treatment for 2 weeks (F_(1,83)_ ​= ​29.8, p ​< ​0.001). After 24 weeks of treatment, improvement in immediate memory was also observed in active groups, compared with sham groups (F_(1,83)_ ​= ​29.7, p ​< ​0.001).

### Predictions of subsequent response to rTMS using early efficacy

We then tested whether these improvements correlated with changes from baseline to follow-up in immediate memory. The results revealed that in the active rTMS group, improvements in immediate memory at week 2 were positively associated with improvements at week 24 (r ​= ​0.407, p ​= ​0.002). Further linear regression analysis showed that efficacy at week 2 could predict the efficacy at week 24 after the treatment with active rTMS, after controlling for age, educational levels, smoking status, disease duration, onset age, and times of hospitalization (β ​= ​0.461, t ​= ​3.322, p ​= ​0.002).

## Discussion

We compared the efficacy of the intervention with 20 ​Hz, 10 ​Hz, and sham rTMS applied to the l-DLPFC on cognitive functions in patients with SZ. 20 ​Hz rTMS intervention, but not 10 ​Hz, showed greater improvements in immediate memory over the 6 weeks of treatment, compared with sham rTMS. At 24-week follow-up, 10 ​Hz and 20 ​Hz rTMS interventions had a delayed effect on immediate memory. Also, the improvements at week 2 could successfully predict the subsequent efficacy of the rTMS interventions at week 24 follow-up.

Updated guidelines on the therapeutic use of rTMS have indicated higher efficacy in the areas of depression, pain, and post-acute motor stroke [45]. However, there is a lack of treatment guidelines for the clinical use of rTMS for treating cognitive deficits in SZ, due to the limited evidence in the field. Our findings are similar to a previous study in patients with SZ with the same treatment protocols, except for the duration of treatment (8 weeks), which found that improvement in immediate memory was only present in the 20-Hz rTMS group for short-term interventions compared with the sham group, and both the 10 ​Hz and 20 ​Hz rTMS were effective for follow-up [33]. This study differed from the previous study in terms of treatment duration and assessment time points. Additionally, in line with our findings, Barr et al. found that 4-week, bilateral, 20-Hz rTMS targeting the l-DLPFC demonstrated a significant beneficial effect on working memory measured by 3-back tasks [35]. The underlying mechanism of therapeutic efficacy of rTMS on cognitive performance may be the fact that it alters cortical excitability, the brain's metabolic activity, neurotransmitters and their receptor expression, neurophysiological activity, neuronal plasticity, local functional connectivity among different brain regions [23,24,46,47].

Notably, we found no difference in efficacy between the 10 ​Hz and sham group at week 6, but significant at week 24, suggesting a delayed effect. While, for 20-Hz rTMS intervention, the improvements were significant both at week 6, and week 24 as compared to sham intervention. This finding was consistent with a meta-analysis of Alzheimer's disease (AD), which reported that 20-Hz rTMS appeared to be more effective in improving cognition than 10-Hz or 1-Hz rTMS in patients [48], suggesting the importance of choosing the appropriate frequency. Young animal model studies of AD also supported that higher frequency rTMS stimulation (e.g. 15 ​Hz rTMS) induced higher expression in H1a and H1b and improved learning compared to 10 ​Hz and sham rTMS stimulation, depending on H1a-dependent functional facilitation [49]. Thus, we speculated that the different cognitive outcomes between the 10 ​Hz and 20 ​Hz groups may be due to the dose-dependent effects of the different stimulation frequencies they have received on cognitive performances.

Furthermore, we found a delayed therapeutic response to active rTMS on immediate memory in patients with SZ. Although long-term follow-up after active rTMS interventions is rarely studied, our findings were consistent with some previous studies in other groups [[50], [51], [52], [53]], and even in our group [36]. For example, RCT trials in SZ reported that the therapeutic effect of rTMS over L-DLPFC for 1 month on negative symptoms may be delayed to week 8, while symptoms remained unchanged in the sham group [54]. Moreover, a similar delayed therapeutic effect of rTMS on auditory hallucination has been reported in patients with SZ [55]. The beneficial effects of active rTMS on immediate memory may be partly related to the regulation of brain metabolic activity, expressions of neurotransmitters and their receptors and neuronal plasticity, which does take time. On the other hand, the left DLPFC is also the rTMS target for treating treatment-resistant major depressive disorders [56]. Reductions in depressive symptoms have been shown to be related to cognitive improvements in various disorders. For example, Baer et al. reported that remitters improved cognition significantly more than non-remitters [57]. Therefore, it is speculated that rTMS over left DLPFC might improve the depressive symptoms of those patients with SZ in this study, which further led to cognitive improvements. In contrast, some studies of the treatment of cognitive functions in depression disorders reported that improvements in self-reported cognitive functioning were not significantly correlated with changes in depressive symptoms [58]. McIntyre et al., also reported that depressed individuals who achieved clinical remission did not improve more on cognition than individuals who did not achieve remission [59]. However, in this study, the depressive symptoms were not measured in patients, and we cannot evaluate if the depressive symptoms were improved and how they were associated with the improvement in cognitive functions. Further research with longer follow-ups is warranted to determine the therapeutic effect of rTMS on depressive symptoms and cognitive performances in SZ.

Our study also demonstrated that the efficacy at week 2 successfully predicted the subsequent efficacy of rTMS interventions at follow-up. Previous studies have shown that early improvements at 10 sessions may be a predictor for rTMS intervention outcomes in major depression [60]. Conversely, another study provides evidence that patients with depression who did not show a response to 20 treatments of rTMS may convert to responders when the treatment course is extended beyond 20 sessions [61]. Our study is the first to report early efficacy prediction of late response to rTMS in SZ. There is some evidence that improvements in immediate memory were associated with reductions in clinical symptoms in SZ [62,63] and that symptomatic and cognitive changes were not independent, but interact. Consistent with other studies [58,59], our study showed significant improvements in cognitive functions at follow-up without significant changes in the severity of clinical symptoms assessed by the PANSS scale. Our findings may exert a great seminal impact on clinical practice and thus switch treatment strategies for those who do not respond to rTMS. The discrepancies regarding the therapeutic effects of rTMS for cognitive functions of SZ may be related to patient characteristics. Currently, SZ patients cannot be effectively categorized into responders and non-responders. Early improvements in cognitive performances that predict long-term outcomes could provide important clinical implications to help improve the early recognition of adverse outcomes, which is worth investigating further.

However, this study found that rTMS showed improvement only in immediate memory, but not in other cognitive functions (e.g., attention). rTMS targeted at l-DLPFC in the current study, which is well-known for its role in maintaining and manipulating information in short-term memory, cognitive flexibility, and inhibitory control [[64], [65], [66], [67], [68]]. The DLPFC also plays a role in top-down and goal-directed attentional control to help individuals focus their attention [69]. The possible explanation for why rTMS showed no effect on other domains of cognitive functions in SZ might be related to the sensitivity of cognitive assessment tools, duration and frequency of rTMS treatment, heterogeneity of cognitive deficits in SZ, and patient expectations of cognitive tasks.

It is worth noting that there were significant differences in terms of demographics and cognitive performances between completers (n ​= ​63) and dropouts (n ​= ​21) in our study, which may introduce selection bias. Considering the similar number of dropouts among the three groups, it is speculated to have little potential impact on the results of this study. Completers may represent a group of patients with good adherence to the treatment protocol. Non-completers may drop out of the study due to side effects, lack of perceived efficacy, or other personal reasons. The distinction between these two groups has interesting insights into the efficacy of the treatment, patient compliance, and factors that influence the success of rTMS for SZ. Identifying predictors of completion could help develop strategies to improve the adherence and effectiveness of rTMS for SZ, which could inform both clinical practice and future research directions. Our study provides evidence that younger patients with a shorter duration of illness and better cognitive performances were likely to complete the course of rTMS treatment.

### Limitations

Several limitations study must be considered. The first limitation was the relatively small sample sizes. We only found a benefit of rTMS on immediate memory, and the negative findings of the beneficial effect on other cognitive domains of SZ might be partly associated with low statistical power. Our findings need to be replicated in a larger sample of patients with SZ. Second, we recruited only male veterans with SZ because the majority of patients at our hospital were male. Thus, these findings cannot be generalized to female patients, which weakens the generalization of the findings in clinical practice. Third, cognitive functions were assessed using the RBANS scale. However, it is not designed to be sensitive to cognitive deficits in SZ, although it is used as a reliable screening scale for cognitive deficits. In particular, it is unable to capture the executive functions, which have been revealed to be correlated with DLPFC. Fourth, this study did not directly investigate the potential mechanism underlying the therapeutic effect of active rTMS on cognitions. Further research was needed to explore the underlying mechanisms through which rTMS shows an effect on immediate memory in SZ. Fifth, cognitive performances were assessed using the RBANS, which was used to measure only five domains of cognitive functions. We did not use other cognitive assessing tools, for example, the MATRICS Consensus Cognitive Battery, built explicitly for evaluating cognitive deficits of SZ and improvements in clinical trials. Although RBANS has been reported to be a reliable tool for cognitive deficits, it is not sensitive to cognitive deficits specific to SZ, especially in dimensions e.g. executive functions. Sixth, individuals in the sham stimulation group also had improved immediate memory scores at the end of treatment, due to the placebo effect in rTMS interventions. This finding was consistent with a prior review in SZ [70]. It might be a major source of bias in the assessment of rTMS intervention efficacy. Therefore, the results of our study should be interpreted with caution. Seventh, we acknowledge a potential limitation to combining the outcomes of 10 ​Hz and 20 ​Hz stimulation frequencies in the current study. It is important to note that the combination of these two frequencies may not be straightforward, as it may mask information about the efficacy of individual stimuli and results need to be interpreted with caution.

In conclusion, this preliminary trial aimed at exploring the effects of rTMS on cognitive ability reveals that rTMS stimulation was beneficial for immediate memory in patients with SZ, and has a delayed effect. Subgroup analysis showed that 20-Hz rTMS produced greater improvements compared with 10-Hz rTMS at week 6. Our study indicates that rTMS is a beneficial treatment option for improving cognitive deficits in SZ. However, given the small sample size, these findings should be interpreted with caution. Further large RCT trials are needed to verify the therapeutic beneficial effects on cognitive performances in patients with SZ.

## Authors’ Contributions

ZY and YH were responsible for study design, statistical analysis, and manuscript preparation. ZY, XG, FW and MX were responsible for recruiting the patients, performing the clinical rating and collecting the clinical data. MX and YH were involved in evolving the ideas and editing the manuscript. FW and YH were involved in writing the protocol and co-wrote the paper. All authors have contributed to and approved the final manuscript.

## Declaration of competing interest

All authors declare that they have no conflict of interest.

## Funding

This study was funded by Key-Area Research and Development Program of Guangdong Province (2023B0303020001), 10.13039/100014717National Natural Science Foundation of China (82301688), Science and Technology Program of Guangzhou (202206060005, 202201010093, 2023A03J0856), Guangdong Basic and Applied Basic Research Foundation Outstanding Youth Project (2021B1515020064), Medical Science and Technology Research Foundation of Guangdong (A2023224), Primary and Secondary School Teachers' educational Capacity Improvement Program of Guangdong (2023YQJK438), Health Science and Technology Program of Guangzhou (20231A010036), and Guangzhou Research-oriented Hospital. The authors report no biomedical financial interests or potential conflicts of interest.

## Ethics Approval

This study was approved by the Institutional Review Board of HeBei Province Hospital.

## Acknowledgments

We thank all participants in this study for their contributions.

## Consent to Participate

Written informed consent was obtained from all participants.

## Availability of Data and Materials

The data that support the findings of this study are available on request from the corresponding author. The data are not publicly available due to privacy or ethical restrictions.
